# Intervertebral disc cell chondroptosis elicits neutrophil response in *Staphylococcus aureus* spondylodiscitis

**DOI:** 10.3389/fimmu.2022.908211

**Published:** 2022-07-28

**Authors:** Tiziano A. Schweizer, Federica Andreoni, Claudio Acevedo, Thomas C. Scheier, Irina Heggli, Ewerton Marques Maggio, Nadia Eberhard, Silvio D. Brugger, Stefan Dudli, Annelies S. Zinkernagel

**Affiliations:** ^1^ Department of Infectious Diseases and Hospital Epidemiology, University Hospital Zurich, University of Zurich, Zurich, Switzerland; ^2^ Center of Experimental Rheumatology, University Hospital Zurich and Balgrist University Hospital, University of Zurich, Zurich, Switzerland; ^3^ Department of Physical Medicine and Rheumatology, University Hospital Zurich and Balgrist University Hospital, University of Zurich, Zurich, Switzerland; ^4^ Department of Pathology and Molecular Pathology, University Hospital Zurich, University of Zurich, Zurich, Switzerland; ^5^ Center for Applied Biotechnology and Molecular Medicine (CABMM), University of Zurich, Zurich, Switzerland

**Keywords:** spondylodiscitis, intervertebral disc cells, *Staphylococcus aureus*, cell death, chondroptosis, neutrophils

## Abstract

To understand the pathophysiology of spondylodiscitis due to *Staphylococcus aureus*, an emerging infectious disease of the intervertebral disc (IVD) and vertebral body with a high complication rate, we combined clinical insights and experimental approaches. Clinical data and histological material of nine patients suffering from *S. aureus* spondylodiscitis were retrospectively collected at a single center. To mirror the clinical findings experimentally, we developed a novel porcine *ex vivo* model mimicking acute *S. aureus* spondylodiscitis and assessed the interaction between *S. aureus* and IVD cells within their native environment. In addition, the inflammatory features underlying this interaction were assessed in primary human IVD cells. Finally, mirroring the clinical findings, we assessed primary human neutrophils for their ability to respond to secreted inflammatory modulators of IVD cells upon the *S. aureus* challenge. Acute *S. aureus* spondylodiscitis in patients was characterized by tissue necrosis and neutrophil infiltration. Additionally, the presence of empty IVD cells’ lacunae was observed. This was mirrored in the *ex vivo* porcine model, where *S. aureus* induced extensive IVD cell death, leading to empty lacunae. Concomitant engagement of the apoptotic and pyroptotic cell death pathways was observed in primary human IVD cells, resulting in cytokine release. Among the released cytokines, functionally intact neutrophil-priming as well as broad pro- and anti-inflammatory cytokines which are known for their involvement in IVD degeneration were found. In patients as well as *ex vivo* in a novel porcine model, *S. aureus* IVD infection caused IVD cell death, resulting in empty lacunae, which was accompanied by the release of inflammatory markers and recruitment of neutrophils. These findings offer valuable insights into the important role of inflammatory IVD cell death during spondylodiscitis and potential future therapeutic approaches.

## Introduction

The incidence of *Staphylococcus aureus* spondylodiscitis, an infection of the intervertebral disc (IVD) and adjacent vertebral bodies, has been steadily rising during the last two decades, classifying it as an emerging infectious disease (1, 2). Spondylodiscitis is a severe disease, often resulting in IVD degeneration (3). This leads to drastic impairment of daily activities in the affected patients, resulting in an increase in the proportion of patients on permanent disability pension and permanent work absence (4). *S. aureus* reaches the spine *via* hematogenous seeding during bacteremia, *per continuitatem* by spread from local soft tissue infections or by direct injection, such as unintentional inoculation during medical procedures (5–7). Due to the absence of immune cells and limited vascularization in the IVD, which impairs the penetration of the antibiotics into the IVD, prolonged antibiotic treatment for a minimum of 6 weeks is required in order to treat the bacterial infection (8, 9). However, the pathophysiology of *S. aureus* spondylodiscitis resulting in IVD destruction as well as the potential inflammatory involvement of IVD cells and therefore crucial insights, which would allow to improve treatment, remains still vastly unknown, among others due to the lack of adequate models.

Since animal models of spondylodiscitis are work-intensive and only allow for low throughput and read-outs, we aimed at establishing a porcine *ex vivo* model to assess the initial interaction between *S. aureus* and IVD cells in their native environment. Porcine models are more frequently established as preclinical models in infectious diseases since their anatomical, physiological, and immunological response properties are close to those of humans (10–12). Furthermore, pigs, as well as humans, are frequently colonized with *Staphylococcus* spp. and are among the livestock animals with reported cases of spondylodiscitis, caused predominantly by *Streptococcus* and *Staphylococcus* spp (13).

The human adult IVD contains only three distinct types of IVD cells, which maintain IVD homeostasis, nucleus pulposus cells found in the center, annulus fibrosus cells found in the outer margins of the IVD, and cartilaginous endplate cells at the intersection between the IVD and the vertebral body (14). IVD cells possess phagocytic capacity, allowing them to degrade IVD tissue and remove dead IVD cells but also, as recently shown, to phagocytose live *S. aureus via* the Toll-like receptor 2 (TLR2) pathway (15–17). However, the fate of IVD cells after encountering *S. aureus* still remains unknown. In contrast to the fulminant infection caused by *S. aureus*, the Gram-positive commensal *Cutibacterium acnes* can cause low-grade chronic IVD infections, during which caspase-mediated IVD cell death following TLR2 recognition was reported (18), suggesting a key role for regulated cell death in spondylodiscitis progression.

Regulated IVD cell death has been extensively studied in connection to its potential role in IVD degeneration. Studies showed that in sterile IVD inflammation, IVD cells in both human and porcine spines frequently undergo regulated cell death morphologically similar to chondroptosis (19, 20). Chondroptosis was first described for articular chondrocytes in the growth plate of rabbit femurs (21, 22) and is considered to be an apoptotic-derived regulated cell death type, characterized by the activation of apoptotic caspases and formation of autophagic vacuoles to sequester and degrade cellular material (23). Furthermore, IVD cells have the ability to express and secrete a wide array of cytokines in response to various environmental stimuli (24–27). Cytokine secretion might be enhanced during chondroptosis of IVD cells and potentially cause a strong local immune stimulation (28, 29).

In the present study, we aimed at characterizing the pathophysiology of *S. aureus* spondylodiscitis. We used a multipronged approach, starting with dissection of the clinical and histological presentation of *S. aureus* spondylodiscitis, translating these observations into a newly established porcine *ex vivo* model of spondylodiscitis and finally characterizing molecular changes occurring in primary human IVD cells upon the *S. aureus* challenge as well as the neutrophil recruitment and activation potential of IVD cells. The findings of our study deliver important insights into the pathophysiology of *S. aureus* spondylodiscitis and potential alternative treatment approaches.

## Material and methods

### Ethical requirements

Use of patient-derived IVD material (Business Administration System for Ethics Committees [BASEC] No. 2018-01486) as well as clinical data of spondylodiscitis patients ([BASEC] No. 2016-00145, 2017-02225 and 2017-01140) and isolation of human neutrophils ([BASEC] No. 2019-01735) was done in accordance with the Declaration of Helsinki and approved by the Cantonal Ethical Research Committee Zurich. The medical documentation of nine patients suffering from spondylodiscitis and treated at the University Hospital of Zurich, Switzerland, between 2011 and 2020 was reviewed. The diagnosis of spondylodiscitis was based on microbiological, clinical, and radiological parameters as well as laboratory tests. Porcine spines were obtained from freshly euthanized animals either from a local butcher (Metzgerei Angst, Zurich) or from the Center for Surgical Research at the University Hospital Zurich.

### Histology

Patient biopsies and porcine spine punches were fixed in 4% buffered formalin and paraffin embedded. Sections (2 μm) were stained with hematoxylin and eosin (H&E), Brown–Brenn (BB), Safranin-o, and Fast-green or Gram-staining. For immunohistochemistry (IHC), formalin-fixed paraffin-embedded tissue sections were pre-treated with the BOND Epitope Retrieval Solution 2 (Leica Biosystems) at 100°C for 30 min. They were stained with anti-cleaved caspase-3 antibody (polyclonal, ab2302, abcam) and caspase-1 antibody (polyclonal, ab62698, abcam) for 30 min. For detection, the slides were stained with the Bond Polymer Refine Detection HRP Kit (Leica Biosystems), according to the manufacturer’s instruction, and counterstained with hematoxylin. Whole-slide scanning and photomicrography were performed with a 108NanoZoomer 2.0-HT digital slide Scanner (Hamamatsu, Houston, TX, USA).

### Bacterial strains and growth conditions

*S. aureus* JE2 USA300 (NARSA) and the clinical isolates were maintained on blood agar plates (Columbia agar + 5% sheep blood, BD) and grown in Tryptic Soy Broth (TSB, BD) at 37°C and 220 rpm for 16 h. Cultures were diluted in fresh TSB and grown to an exponential phase for the challenge of IVD cells. For the supernatant challenge, overnight cultures were diluted in fresh Dulbecco’s modified Eagle medium (DMEM)/F12 (Gibco) + 10% fetal calf serum (FCS) and grown to exponential phase (approx. 3 h). The cultures were filter sterilized with a 0.22-μm filter and used in a ¼ dilution.

### Preparation and infection of porcine intervertebral disc punches

Porcine spines were cleaned with 70% EtOH wipes. The IVDs were removed along the endplates on both sides, and annulus fibrosus IVD punches were prepared with a 5-mm biopsy punch (Kai Medical). IVD punches were dissected longitudinally in the center and placed with the IVD side facing upwards in 96-well plates in DMEM/F12 + 10% FCS. IVD punches were infected with bacteria at an inoculum of 4 × 10^5^ colony-forming units (CFUs) and incubated at 37°C + 5% CO_2_.

### Assessment of bacterial growth within intervertebral disc punches

At indicated timepoints, the medium was removed, and the IVD punches were transferred to new wells. They were washed twice with Dulbecco’s phosphate-buffered saline (DPBS) and sonicated for 3 min to remove adhering bacteria. Next, they were placed into tubes with metallic beads and homogenized in a TissueLyser (Qiagen) at 30 Hz for 10 min. The tubes were centrifuged at 1,200 rpm for 3 min after which the supernatant was collected into fresh tubes and centrifuged at 14,000 rpm for 3 min. The resulting pellet was lysed with water, serially diluted, and spotted on TSB agar (TSA) plates.

### Assessment of cell death of intervertebral disc cells within intervertebral disc punches

Washed IVD punches were fragmented into four pieces and enzymatically digested with enzymatic digestion buffer consisting of DMEM/F12 (Gibco), 1 mg/ml of Collagenase II (Thermo Fisher), and 1 mg/ml of Pronase K (Roche) with penicillin/streptomycin (P/S, Gibco) for 30 min at 37°C and 400 rpm. Next, isolated cells were filtered through strainer cap tubes (Falcon), and the remaining red blood cells were subsequently lysed with water. The cells were stained with Annexin V-FITC/7AAD as described previously (30). Cells were acquired on an Attune NxT (Thermo Fisher). To determine purity of isolated IVD cells, they were washed with fluorescence-activated cell sorting (FACS) buffer (DPBS + 5% FCS + 2 mM of EDTA) and stained with 1:50 pig anti-CD31 RPE (clone LCI-4) and pig anti-CD45 AF647 (K252.1E4), both BioRad.

### Transmission electron microscopy

Washed IVD punches were fixed in 2.5% glutaraldehyde at 4°C for 72 h. Next, IVD punches were decalcified with EDTA for 10 days. After decalcification, the samples were stained and processed for image analysis as previously described (31). Images were taken with a 120-kV transmission electron microscope (FEI Tecnai G2 Spirit) equipped with two digital CCD cameras.

### Isolation and infection of human intervertebral disc cells

Human IVD tissue was obtained from a total of 13 patients undergoing surgical intervention due to spinal stenosis or deformity. Multiple levels of surgery per single patient were possible and were as follows: four at L5–S1, one at L1–L2, four at L2–L3, two at L3–L4, and five at L4–L5. The tissue was fragmented mechanically and enzymatically digested overnight at 37°C and 400 rpm. Isolated cells were filtered through strainer cap tubes before seeding in tissue culture flasks (TPP) in DMEM/F12 + 10% FCS + P/S. For experiments, passage 1 cells were seeded in 96-well flat-bottom plates at a density of 1 × 10^5^ cells per well without antibiotics. Infection at multiplicity of infection (MOI) 10 and intracellular survival were carried out as described previously (32).

### Assessment of intervertebral disc cell death

Cells were detached and stained with Annexin V-FITC/7AAD as previously described (30). Cells were acquired on an Attune NxT. If required, pan-caspase (50 μM of Q-VD-OPH, Sigma), caspase-1 (50 μM of Z-YVAD-FMK, Sigma), caspase-8 (50 μM of Z-IETD-FMK, R&D Systems), or caspase-9 inhibitors (50 μM of Z-LEHD-FMK, R&D Systems) were added 30 min prior to bacterial challenge. Controls were treated with dimethyl sulfoxide (DMSO) only.

### Confocal laser scanning microscopy

Challenged or unchallenged IVD cells in 8-well μ slides (ibidi^®^) were fixed with 4% paraformaldehyde (PFA). Next, they were permeabilized by Saponin and stained with 20 μM of Hoechst (Themo Fisher) and 1.5 U Rhodamine Phalloidin (Thermo Fisher). Samples were visualized and acquired by confocal laser scanning microscopy (CLSM) with a Leica TCS SP8 inverted microscope (Leica) under a 63×/1.4 NA oil immersion objective. The obtained images were processed using Imaris 9.2.0 (Bitplane).

### Assessment of caspase activity and membrane permeability

The Caspase-Glo^®^ 3/7, Caspase-Glo^®^ 8, and Caspase-Glo^®^ 9 (all Promega) kits were used as previously described (30). The caspase-1 activity assay kit (Novus Biologicals) and the mitochondrial ToxGlo™ (Promega) assay were used according to the manufacturer’s instructions. Fluorescence (488/525 nm) and luminescence were measured with the SpectraMax i3 (Molecular Devices).

### Cytokine analysis

Cell culture supernatants were filtered through a 0.22-μm filter, and cytokine levels were analyzed on a Luminex™ MAGPIX™ (Thermo Fisher) as previously described (30, 32). Analysis was performed using the xPONENT^®^ software. Data were validated additionally with the ProcartaPlex Analyst software (Thermo Fisher).

### Neutrophil isolation and stimulation

Neutrophils from healthy donors were isolated with the EasySep™ Direct Human Neutrophil Isolation Kit (StemCell Technologies) as previously described (30, 32). Neutrophils were resuspended in DMEM/F12 and counted on an Attune NxT. They were seeded in 96-well V-well canonical plates and stimulated with either fresh medium or supernatant from un- or challenged IVD cells in a 1:3 ratio for 3 h. Neutrophils were seeded at a density of 2.5 × 10^5^ if not indicated differently.

### Cell surface receptor expression

Neutrophils were stained as described previously (32). Cells were stained 1:750 with the Near-IR™ Live/Dead reagent (Thermo Fisher), 1:50 anti-CD15 eFluor450 (clone: HI98), anti-CD181 FITC (8F1-1-4), anti-CD182 PerCP-eFluor710 (5E8-C7-F10), anti-CD183 PE-eFluor610 (CEW33D), and anti-CD66b APC (G10F5) from Thermo Fisher and anti-CD184 BV605 (12G5) from Biolegend. Cells were acquired on an Attune NxT.

### Migration assay

For migration assays, the HTS Transwell^®^-96 well permeable support has a 5-μm pore size polycarbonate membrane (Corning). In the bottom wells, either fresh medium or supernatant from un- or challenged IVD cells was placed in a 1:3 ratio. In the inserts, neutrophils were added at a density of 1 × 10^5^ cells. After 15 and 45 min, the medium in the bottom well was collected and washed with FACS buffer, followed by staining with anti-CD66b for 30 min at 4°C. The absolute number of migrated neutrophils was acquired in 150-μl volume on an Attune NxT.

### Reactive oxygen species production

Reactive oxygen species (ROS) production was assessed after 2.5 h of stimulation as previously described (32) with 5 μM of the CellROX™ green reagent (Thermo Fisher). Cells were acquired on an Attune NxT.

### Statistical analysis

Statistical analysis was done with GraphPad Prism 8. Samples were first assessed for normal distribution and then tested for statistical significance. The applied tests are indicated within each figure legend.

## Results

### Demographic and clinical characteristics of *Staphylococcus aureus* spondylodiscitis

**Figure 1:**
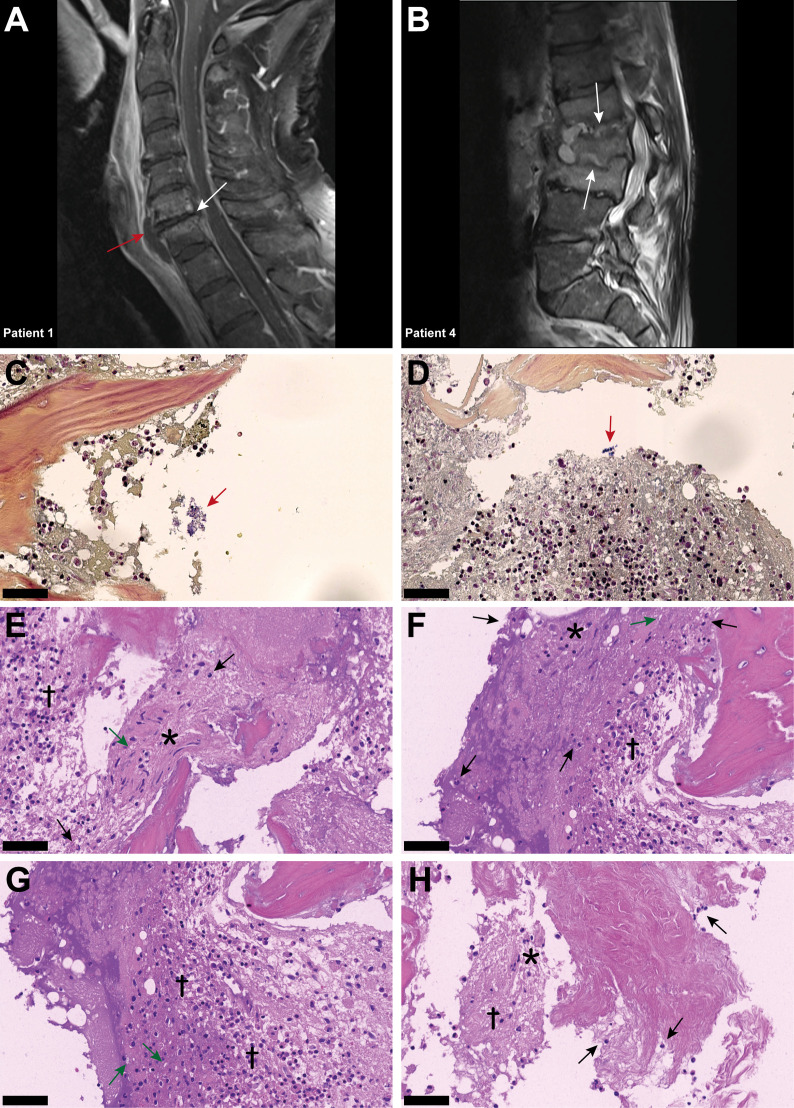
Inflammatory infiltrates with presence of empty IVD cells’ lacunae and neutrophils in IVD during acute *Staphylococcus aureus* spondylodiscitis. **(A, B)** T1-weighted MRI scans showing inflammatory infiltrates into and degenerative changes of the IVD in patients 1 and 4. White arrows indicate localization of spondylodiscitis; red arrow indicates paravertebral abscess. **(C, D)** BB staining of the IVD of patient 3. Red arrow, Gram-positive cocci. **(D–H)** H&E staining of the IVD of patient 3. Cross, inflammation and necrosis; asterisk, IVD cells; green arrow, empty lacunae; black arrow, immune cells (neutrophils). Scale bars indicate 50 μm. BB, Brown–Brenn; H&E, hematoxylin and eosin, IVD, intervertebral disc.

The medical documentation of a case series of nine hospitalized patients suffering from spondylodiscitis at the University Hospital Zurich, Switzerland, with a positive *S. aureus* blood culture was reviewed (**Table 1**). The median age was 58 years. Seven patients had at least one predisposing factor for developing spondylodiscitis, such as diabetes, hemodialysis, old age, history of spinal surgery, cardiac devices, or immunosuppression, while two patients had no identifiable comorbidities. The female to male ratio was 5:4. All patients showed elevated C-reactive protein (CRP) and white blood cell count (WBC) at admission (**Table 2**). The length of hospital stay (LOS) ranged from 10 to 68 days, with a median LOS of 21 days and a 30-day survival rate of 77.8%. The death of the two patients was associated with hypoxemia due to aspiration and sepsis due to endocarditis. Out of the nine patients, six showed radiological signs of intervertebral disc degeneration with edema (**Figures 1A, B**). Histological IVD material was only available for patients 3 and 6.

**Table 1:** Demographic overview of spondylodiscitis patients.

Patient	Gender	Age	Predisposing factors	CCI
1	Female	31	Not present	0
2	Female	47	Not present	0
3	Male	58	Hemodialysis	2
4	Male	50	Intravenous drug use, previous *Staphylococcus aureus* bacteremia	0
5	Male	78	Not present	0
6	Female	43	Hemodialysis	4
7	Female	80	Not present	0
8	Male	69	Cardiac device, diabetes	4
9	Female	71	Not present	0

CCI, Charlson Comorbidity Index.

**Table 2:** Clinical characteristics and laboratory parameters of spondylodiscitis patients at admission.

Patient	Site of acquisition	Source of infection	Bacteremia	Localization	Radiological signs of degeneration	LOS [days]	30-day survival	CRP [mg/L]	WBC [G/L]
1	CA	Unknown	Yes	Th10–Th11	No	19	Yes	167	14.83
2	CA	Unknown	Yes	L2–L3	Yes	22	Yes	403	21.37
3	HC	Hemodialysis catheter	Yes	Th10–Th12	Yes	25	Yes	88	9.32
4	CA	Unknown	No	L1–L3	Yes	10	Yes	196	7.28
5	CA	Unknown	Yes	C6–C7	Yes	15	Yes	487	15.3
6	HC	Unknown	Yes	Th8–Th9	No	23	Yes	86	8.24
7	CA	Unknown	Yes	L3–L4	Yes	21	No^a^	n/a	n/a
8	CA	Device-related infection	Yes	L2–L4	Yes	68	Yes	339	15.07
9	CA	Soft tissue infection	Yes	L3–L4	n/a	20	No^b^	144	31.2

LOS, length of stay; CRP, C-reactive protein; WBC, white blood cell count; CA, community acquired; HC, healthcare related; Th, thoracic, L, lumbar, C, cervical.

a Death due to hypoxemia during aspiration.

b Death due to sepsis with endocarditis.

### Histopathological findings in *Staphylococcus aureus* spondylodiscitis

In patient 3, Brown–Brenn staining identified the scattered presence of aggregated Gram-positive cocci (**Figures 1C, D**). H&E staining showed strongly inflamed and necrotic tissue, characterized by the presence of empty lacunae, an indication of IVD cell death (**Figures 1E, F**). Additionally, neutrophil infiltration in the IVD tissue was observed, indicating an acute infection based on the histological findings (**Figures 1G, H**). In contrast, in the histology sample from patient 6, the inflammatory infiltrate was mostly composed of lymphocytes, with only a few neutrophils present, and no bacteria were observed (**Supplementary Figure 1**), indicating a chronic situation based on the histological findings.

### *Staphylococcus aureus* challenge induces chondroptosis of intervertebral disc cells in the *ex vivo* porcine spondylodiscitis model

**Figure 2:**
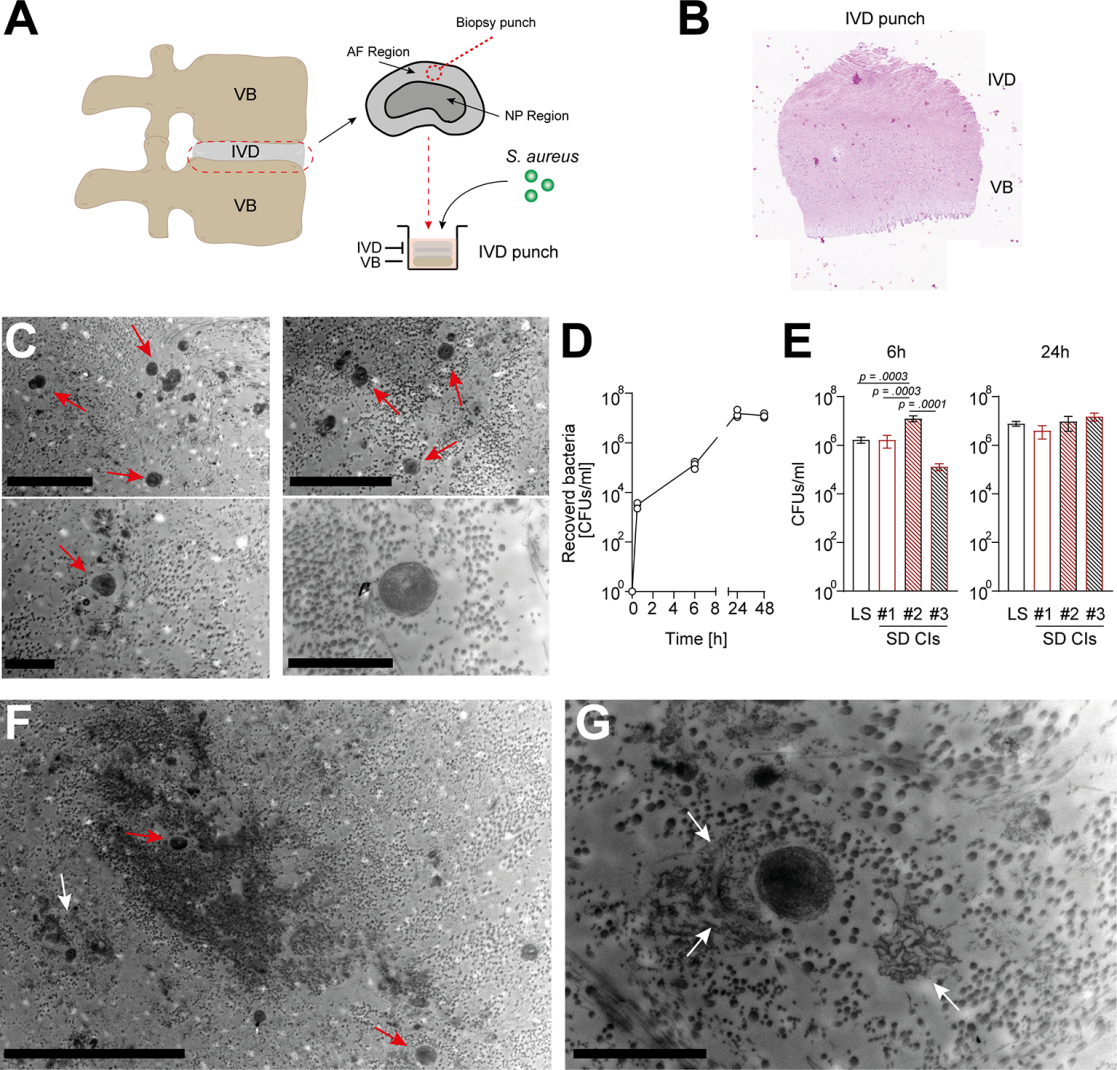
*Staphylococcus aureus* rapidly grows and maintains within a novel porcine *ex vivo* spondylodiscitis model. **(A)** Schematic depiction of porcine spondylodiscitis model set up. IVDs were removed from porcine spines, and disc punches from the AF region were prepared to be inoculated with medium only or *S. aureus* challenge. **(B, C)** H&E staining showing sagittal section of prepared IVD punches. **(C)** TEM of infected IVD punch. **(D)** Growth curve of the *S. aureus* laboratory strain (LS) within the IVD punches over 48 h. **(E)** Comparison of the ability of different *S. aureus* spondylodiscitis (SD) isolates to colonize and grow within the IVD punches. **(F, G)** TEM overview **(F)** and zoomed-in view **(G)** of infected IVD punch. Red arrow, multiple staphylococci; white arrow, lysed IVD cell. Data are presented as mean ± standard deviation from three biological replicates. Statistical analysis was done by one-way ANOVA and Turkey’s multiple comparisons test. Scale bars indicate 5 and 2 μm (**C**, top and bottom panels, respectively), 10 μm **(F)**, and 2 μm **(G)**. IVD, intervertebral disc; AF, annulus fibrosus; NP, nucleus pulposus; H&E, hematoxylin and eosin; TEM, transmission electron microscopy.

To assess a potential direct interaction between *S. aureus* and IVD cells in their native environment, we established a novel porcine *ex vivo* model (**Figures 2A, B**). Since IVD cells found in the porcine nucleus pulposus might still resemble a stem cell-like population, i.e., notochordal cells (33), which are only found in human infants but not in adults, we only selected areas from the annulus fibrosus. The IVD punches were challenged with a patient-derived *S. aureus* strain, to assess whether *S. aureus* could grow and persist in the IVD tissue environment. Transmission electron microscopy (TEM) and histology confirmed the presence of *S. aureus* deeply embedded within the IVD punch (**Figure 2C;**, **Supplementary Figure 2**). *S. aureus* grew within the IVD environment for up to 48 h (**Figure 2D**). When comparing laboratory and different clinical strains (**Supplementary Table 1**), differences in the potential of initial attachment to the IVD and growth within were observed (**Figure 2E**). Of note, staphylococci were also identified within (**Figure 2F**) and close to lysed IVD cells, surrounded by cellular matrix (**Figure 2G**), an indication of *S. aureus* cytotoxicity towards IVD cells.

**Figure 3:**
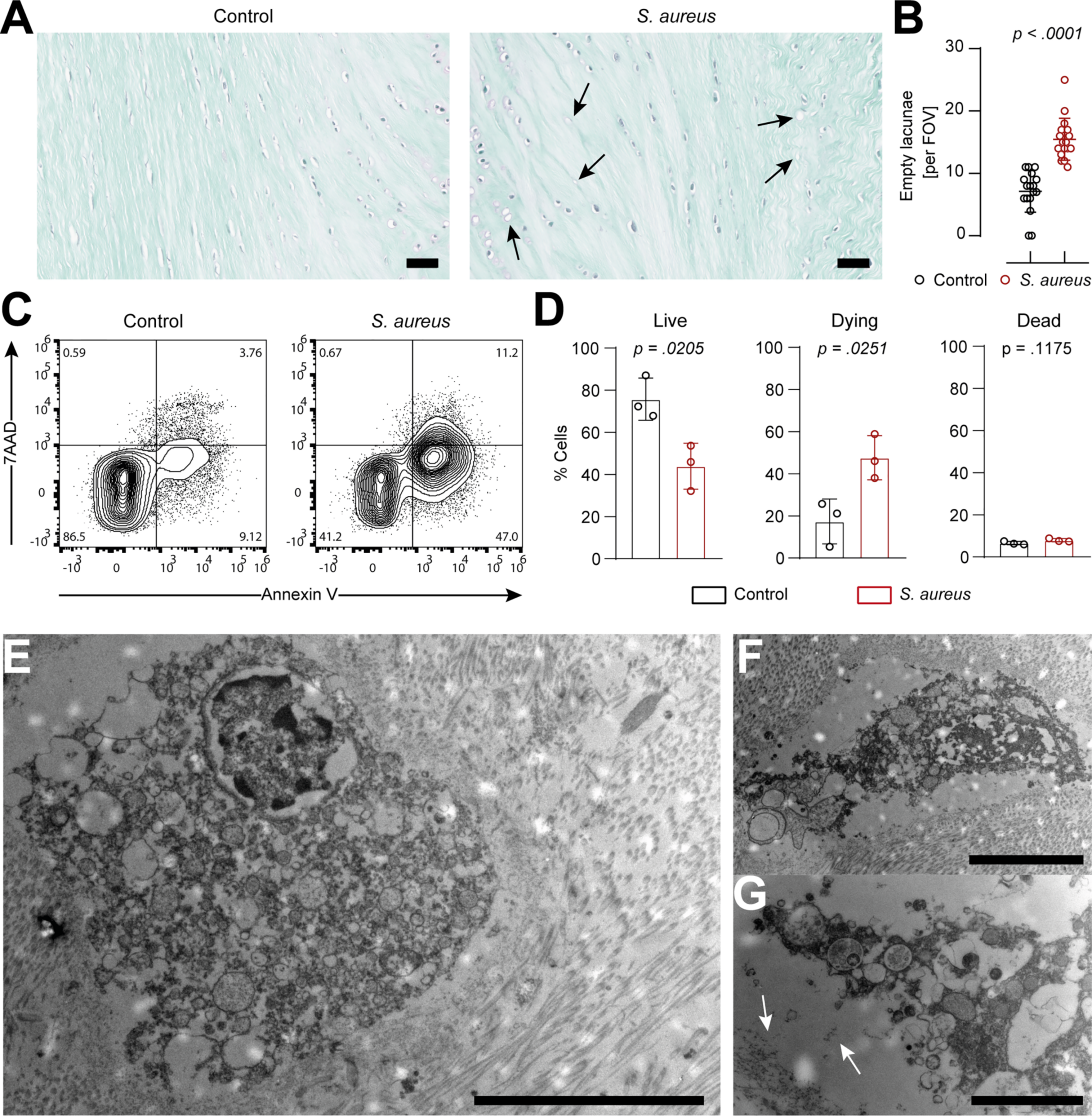
IVD cells in the porcine ex *vivo* model undergo extensive chondroptosis upon *Staphylococcus aureus* challenge. **(A, B)** Safranin-o/fast-green staining of histological slides **(A)** and quantification of empty lacunae in IVD punches either unchallenged or challenged with the clinical *S. aureus* isolate from patient 7 for 24 h. Dark arrows, empty lacunae. In total, 17 randomly selected FOVs from six different IVD punches per group were chosen to evaluate the presence of empty lacunae. **(C, D)** Representative flow cytometry plot of isolated IVD cells from either unchallenged or *S. aureus* challenged IVD punches and stained with Annexin V/7AAD **(C)** and quantification of live (Annexin V^−^, 7AAD^−^), dying (Annexin V^+^, 7AAD^−^), and dead (Annexin V^+^, 7AAD^+^) IVD cells **(D)**. **(E–G)** TEM of IVD cells within *S. aureus* challenged IVD punches. White arrows, deposition of cellular material to the edge of the lacuna. In panel **(D)**, data are presented as mean ± standard deviation from three biological replicates, where each dot represents one biological replicate. Statistical analysis was done by paired t-test. Scale bars indicate 100 μm **(A)**, 10 μm **(E)**, 5 μm **(F)**, and 2 μm **(G)**. IVD, intervertebral disc; FOV, field of view; TEM, transmission electron microscopy.

Histological analysis showed an increase in the presence of empty lacunae in IVD punches challenged with *S. aureus*, an indication of IVD cell death (22) (**Figures 3A, B**). In order to understand whether IVD cells underwent regulated cell death, IVD cells isolated from IVD punches challenged with *S. aureus* or left unchallenged were assessed by flow cytometry using the Annexin V/7AAD staining method (**Supplementary Figure 3A**). The isolated IVD cells were very pure and showed no signs of contamination by either immune or endothelial cells (**Supplementary Figure 3B**). IVD cells isolated from the *S. aureus* challenged IVD punches showed a significantly lower proportion of viable cells and a higher proportion of cells undergoing regulated cell death as compared to IVD cells isolated from uninfected IVD punches (**Figures 3C, D**). Morphological ultrastructural analysis by TEM revealed the loss of the usually elongated fibroblastic phenotype of annulus fibrosus IVD cells when challenged with *S. aureus* (**Figure 3E**; **Supplementary Figure 3C**). Nuclei showed signs of local degradation but no dispersion in the cellular lumen. Furthermore, membrane integrity seemed to be lost, and many vacuoles were observed (**Figures 3E, F**). Additionally, cellular debris was located at the border of the lacunae (**Figure 3G**).

### *Staphylococcus aureus-*induced chondroptosis in human intervertebral disc cells is linked to apoptotic and pyroptotic caspases

**Figure 4:**
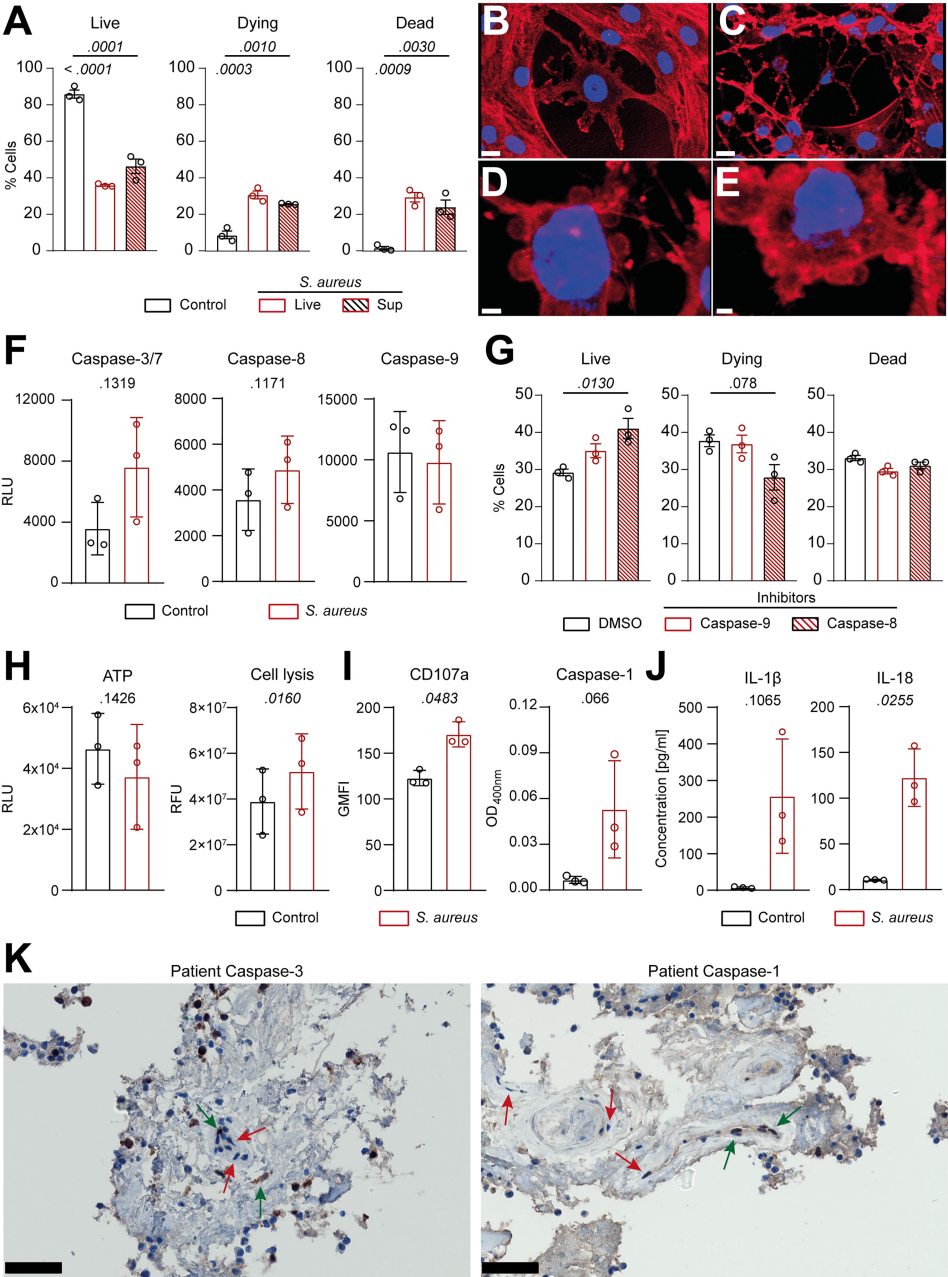
Human primary IVD cells undergo chondroptosis linked to concomitant activation of the apoptotic and pyroptotic pathway upon *Staphylococcus aureus* challenge. **(A)** Isolated human primary IVD cells were infected with the clinical *S. aureus* isolate from patient 7 at MOI 10 or *S. aureus* supernatant for 24 h, and viability was assessed by flow cytometry with Annexin V/7AAD, quantifying live (Annexin V^−^, 7AAD^−^), dying (Annexin V^+^, 7AAD^−^), and dead (Annexin V^+^, 7AAD^+^) IVD cells. **(B–E)** Representative CLSM images showing an overview of unchallenged IVD cells **(B)** and IVD cells challenged with live *S. aureus* **(C–E)**. Cells were stained with Hoechst (blue, nucleus) and Rhodamine-phalloidin (red, actin cytoskeleton). **(F)** Analysis of caspase-3/7, caspase-8, and caspase-9 activity in unchallenged or challenged IVD cells. **(G)** Flow cytometry analysis of challenged IVD cells in the presence or absence of caspase-8 or caspase-9 inhibitors (50 μM), quantifying live, dying, and dead cells. **(H, I)** Analysis of ATP levels, cell membrane integrity, and marker for lysosomal exocytosis (CD107a) **(H)** and caspase-1 activity **(I)** in unchallenged or challenged IVD cells. **(J)** Luminex-based analysis of IL-1β and IL-18 secretion into supernatant by unchallenged or challenged IVD cells. **(K)** IHC of cleaved caspase-3 and caspase-1 in the IVD of patient 3. Green arrow, positive IVD cells; red arrow, negative IVD cells. Data are presented as mean ± standard deviation from three biological replicates, where each dot represents one biological replicate. Statistical analysis was done by one-way ANOVA and Turkey’s multiple comparisons test or paired t-test. Scale bars indicate 15 μm **(B, C)**, 3 μm **(D)** 2 μm **(E)**, and 50 μm **(K)**. IVD, intervertebral disc; MOI, multiplicity of infection; CLSM, confocal laser scanning microscopy; IHC, immunohistochemistry.

To better understand the molecular mechanisms underlying the interaction between *S. aureus* and IVD cells, we assessed the response of primary human annulus fibrosus IVD cells to the *S. aureus* challenge. Isolated primary human IVD cells (**Supplementary Figures 4A, B**) efficiently phagocytosed but failed to completely eradicate intracellular *S. aureus*, over an infection period of 3 days (**Supplementary Figures 4C, D**). Challenge with either *S. aureus* or *S. aureus* conditioned-medium caused IVD cells to undergo regulated cell death (**Figure 4A**; **Supplementary Figure 4E**). Visual assessment by confocal laser scanning microscopy revealed altered IVD cell morphology (**Figures 4B, C**) resembling the hallmarks of chondroptosis, such as membrane blebbing and the presence of vacuoles with intact nuclei, when challenged with *S. aureus*, which was not observed in unchallenged IVD cells (**Figures 4D,E**). Next, we assessed whether caspases were involved in *S. aureus-*induced chondroptosis. Indeed, we found the elevated activity of the effector caspase-3/7 as well as the initiator caspase-8 (**Figure 4F**), whereas no changes in caspase-9 activity were observed. Caspase-8 inhibition led to significantly enhanced survival, while caspase-9 inhibition did not block cell death induction, further confirming our findings (**Figure 4G**). Since we previously observed that porcine IVD cells presented with a loss of membrane integrity, we also investigated whether chondroptosis upon the *S. aureus* challenge involved changes to the membrane permeability of primary human IVD cells. Even though no significant differences in cellular ATP levels were found, the *S. aureus* challenge led to an increased loss of membrane integrity (**Figure 4H**). The observed vacuoles might represent lysosomal exocytosis, i.e., for transport of cytokines to the extracellular space. Therefore, we assessed CD107a expression on IVD cells as a marker for lysosomal exocytosis. We found significantly higher expression of CD107a on IVD cells challenged with *S. aureus* as compared to uninfected cells (**Figure 4I**). Furthermore, we observed increased caspase-1 activity in infected IVD cells (**Figure 4I**), which was further accompanied by IL-1β and IL-18 secretion (**Figure 4J**). Moreover, pan-caspase inhibition showed a significantly higher effect on IVD cell survival than either caspase-1 or caspase-8 inhibition alone (**Supplementary Figure 4F**). Finally, IHC of patient histology showed positive staining for cleaved caspase-3 and caspase-1 in IVD cells during *S. aureus* spondylodiscitis (**Figure 4K**).

### Intervertebral disc cells secrete functional neutrophil-priming cytokines upon *Staphylococcus aureus-*induced chondroptosis

**Figure 5:**
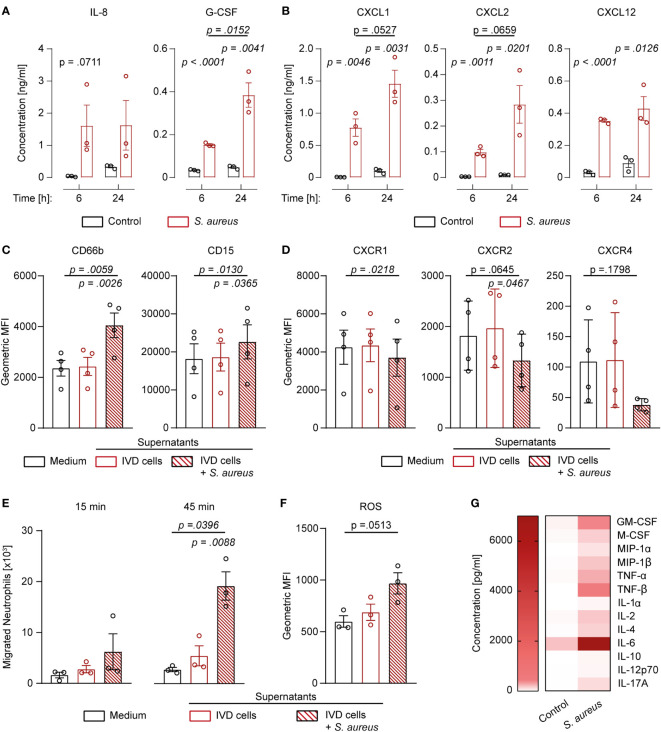
Human IVD cells secrete a broad range of cytokines upon *Staphylococcus aureus* challenge, including functional neutrophil-priming cytokines. **(A, B)** Luminex-based analysis of IL-8, G-CSF **(A)**, as well as CXCL1, CXCL2, and CXCL12 **(B)** secretion into supernatant by unchallenged or *S. aureus-*challenged IVD cells with the clinical *S. aureus* isolate from patient 7. **(C, D)** Activation and maturation marker expression **(C)** and chemokine receptor expression **(D)** on primary human neutrophils stimulated with medium only or cell culture supernatant from either unchallenged or challenged IVD cells. **(E)** Migration assays of primary human neutrophils towards medium only or cell culture supernatant from either unchallenged or challenged IVD cells. **(F)** Assessment of ROS production in primary human neutrophils stimulated with medium only or cell culture supernatant from either unchallenged or challenged IVD cells. **(G)** Luminex-based analysis of broad pro- and anti-inflammatory cytokine secretion into supernatant by unchallenged or challenged IVD cells. Data are presented as mean ± standard deviation from at least three biological replicates; each dot represents one biological replicate. Statistical analysis was done by two-way ANOVA and Sidak’s multiple comparisons test or one-way ANOVA and Turkey’s multiple comparisons test. IVD, intervertebral disc; ROS, reactive oxygen species.

Since challenged IVD cells secreted the pro-inflammatory cytokines IL-1β and IL-18, we investigated whether they also secreted neutrophil-targeting cytokines. *S. aureus* challenged IVD cells secreted significantly elevated levels of IL-8, G-CSF, CXCL1, CXCL2, and CXCL12 (**Figures 5A, B**). Apart from IL-8 and CXCL12, the levels of secreted cytokines were higher at later timepoints of infection. To assess whether the secreted cytokines were functional, isolated primary human neutrophils were stimulated with the filtered supernatant of infected or uninfected IVD cells. Neutrophils stimulated with supernatant from infected IVD cells showed significantly higher expression of CD66b and CD15 as compared to neutrophils stimulated with supernatant from uninfected IVD cells or medium only (**Figure 5C**; **Supplementary Figures 5A–C**). Furthermore, the corresponding neutrophil receptors, CXCR1 for IL-8, G-CSF, and CXCL1, and CXCR2 for IL-8 and CXCL2 as well as CXCR4 for CXCL12, showed decreased expression upon stimulation with supernatant from challenged IVD cells, pointing towards active engagement of these receptors (**Figure 5D**) (34). This was only observed upon stimulation with supernatant from IVD cells at late infection stages, while an early infection stage supernatant did not yield the same effect, making bacterial toxins as a possible bias unlikely (**Supplementary Figure 5D**). We found that significantly more neutrophils migrated towards supernatants from infected IVD cells as compared to supernatants from uninfected IVD cells or medium alone (**Figure 5E**). They also produced higher intracellular reactive oxygen species (ROS) levels (**Figure 5F**). Finally, we also explored the potential of IVD cells to secrete additional cytokines, involved in a more comprehensive immune response. IVD cells secreted a broad range of cytokines upon the *S. aureus* challenge, such as the general inflammation and damage markers IL-1α, IL-6, TNF-α, and TNF-β; the monocyte-stimulating cytokines GM-CSF, M-CSF, MIP-1α, and MIP-1β; the T cell-stimulating cytokines IL-2, IL-12p70, and IL-17A; as well as the anti-inflammatory cytokines IL-4 and IL-10 (**Figure 5G**).

## Discussion

In patients suffering from *S. aureus* spondylodiscitis, we identified the presence of empty lacunae, an indication of IVD cell death, and the influx of neutrophils into the IVD. These findings were mirrored experimentally, by establishing a novel porcine spondylodiscitis model and by using primary human IVD cells. We confirmed that IVD cells underwent chondroptosis, which was linked to a strong immune-stimulating cytokine secretion profile, especially directed towards the activation and recruitment of neutrophils.

In our case series analysis of patients suffering from *S. aureus* spondylodiscitis, we observed increased inflammation as well as IVD degeneration as reported previously for spondylodiscitis (35–37). In the reported cases, the CRP concentration was approximately two- to fourfold elevated in about 95% of spondylodiscitis patients, and roughly one-third of patients displayed mild leukocytosis, similar to our observations (38–40). Usually, patients with spondylodiscitis have at least one predisposing risk factor such as diabetes, hemodialysis, older age, history of spinal surgery, presence of cardiac devices, or immunosuppression (38, 39, 41). Seven out of nine of our patients presented with identifiable risk factors: three patients were older, two patients underwent hemodialysis, one patient had an implanted cardiac device as well as diabetes, and one patient had previously suffered from *S. aureus* bacteremia due to intravenous drug injection. For the first time, we show Gram-positive cocci together with the presence of empty IVD cells’ lacunae, which was not described in the literature so far. This clinical observation shows the interaction between IVD cells and Gram-positive cocci in human IVD.

In order to investigate these clinical findings in detail, we developed a novel porcine *ex vivo* spondylodiscitis model. Of note, IVD cells found in the porcine nucleus pulposus might still resemble a stem cell-like population, which is only found in humans during their infancy (42). Therefore, we used IVD punches prepared exclusively from the annulus fibrosus of porcine IVDs.

*S. aureus* was able to grow to high densities and persist within the IVD environment, as previously reported for other bacteria (18, 25, 42). The observed differences in initial adherence to the IVD tissue between the various clinical bacterial isolates might be explained by the presence or expression of specific virulence factors. *S. aureus* contains many different virulence factors favoring adherence to specific tissues (43, 44). In the case of the collagen-rich IVD matrix, potentially involved adherence factors might be the collagen-binding adhesin, bone sialoprotein, or the fibronectin-binding protein (45–49). However, conflicting results regarding the importance of especially collagen-binding adhesins in the initiation of bone and joint infections exist (50). Furthermore, these adherence factors are redundant and show affinity to various matrix substrates (51). Therefore, an assessment and comparison of the presence of certain adherence factors between the clinical bacterial isolates do not allow to draw a conclusion on their virulence capacity (52). The combination of genetic and phenotypic approaches using a large collection of bacterial isolates or the use of isogenic mutants would be required in order to determine the potentially crucial role of specific bacterial adherence factors.

Challenge with *S. aureus* led to the accumulation of empty lacunae within the IVD in the porcine *ex vivo* model, an indication for regulated IVD cell death (23, 53), which was confirmed by Annexin V staining. However, since Annexin V staining is just an indicator for any type of regulated cell death, it is crucial to further determine the involved regulated cell death pathways (54). Since chondroptosis is regarded as the *de facto* regulated IVD cell death phenotype (20, 23), it is not surprising that IVD cells underwent chondroptosis upon the *S. aureus* challenge, as corroborated by TEM, especially since it was previously observed that IVD cells underwent caspase-mediated regulated cell death upon *C. acnes* challenge (18). One can assume that chondroptosis might be a conserved response of IVD cells towards bacterial encounters.

Chondroptosis was also observed in primary human IVD cells upon the *S. aureus* challenge. Importantly, supernatants of staphylococcal cultures were sufficient to induce chondroptosis, potentially attributable to the presence of secreted toxins. Among them, the staphylococcal α-toxin, a potent inducer of cell death in many different cell types, including articular chondrocytes, might play a key role in inducing chondroptosis in IVD cells (55–57). Furthermore, also the engagement of TLR2 by bacteria-associated molecular patterns might be sufficient to induce chondroptosis in IVD cells (18).

We observed that chondroptosis of human IVD cells upon the *S. aureus* challenge was linked to increased caspase-3/7 and caspase-8 activity, but not caspase-9 activity. Caspase-3/7 activation was previously observed in IVD cells during IVD degeneration (58–61). Both caspase-8 and caspase-9 activation were shown to occur in IVD cells during sterile IVD inflammation (62, 63). However, in our study, IVD cells only showed increased caspase-8 but not caspase-9 activity, suggesting that chondroptosis of IVD cells during the *S. aureus* challenge involves auto- and paracrine signaling of ligands to death receptors, such as the signaling of TNF-α to the TNF I receptor, which might be involved in IVD cell chondroptosis (64, 65). Furthermore, we found that chondroptosis upon the *S. aureus* challenge involved cell membrane integrity loss and lysosome formation. Lysosomal exocytosis is a known mechanism of pro-inflammatory cytokine secretion, best known for IL-1β and IL-18 secretion (28, 29). Both IL-1β and IL-18 are stored within the cell in an inactive pro-form, which needs to be cleaved and activated by proteolytic enzymes, such as caspase-1 (66). This suggests that also caspase-1, which indeed showed increased activity, plays an important role in chondroptosis upon the *S. aureus* challenge. Our findings of IL-1β and IL-18 secretion together with elevated caspase-1 activity in chondroptotic IVD cells were in line with findings of the engagement of the NLRP3/caspase-1 axis in IVD cells accompanied by IL-1β secretion in IVD degeneration (24, 67–70). The IHC findings of the patient spondylodiscitis, although depicting a situation during the course of the disease rather than at the onset, further corroborated the experimental findings of the concomitant involvement of apoptotic and pyroptotic caspases. It was recently shown that the regulated cell death pathways apoptosis, necroptosis, and pyroptosis are intermittently linked and regulated together, which was termed PANoptosis (71). Given our findings of concomitant activation of apoptotic and pyroptotic caspases, chondroptosis seems to follow a conserved pattern of regulated cell death activation, relying on multiple initiation and execution platforms.

Previous research showed that IVD cells express and secrete neutrophil-priming cytokines upon IL-1β stimulation (72, 73). We further corroborated these findings in the context of the *S. aureus* challenge of IVD cells and showed that the secreted neutrophil-targeting cytokines were functionally intact, leading to neutrophil priming. However, whether neutrophil recruitment and activation in *S. aureus* spondylodiscitis are beneficial to clear the infection or detrimental, resulting in tissue damage and further inflammation, still needs to be elucidated, since extensive ROS release causes tissue injury (74). Nevertheless, these findings add to the growing body of evidence that IVD cells are potent immune cells’ recruiter, as previously shown for T cells *via* the secretion of CCL20 from the IVD (26). Apart from neutrophil or T cell-specific cytokines, various studies showed that the pro-inflammatory cytokines TNF-α, TNF-β, IL-1α, IL-1β, IL-6, and IL-17A, also secreted after the *S. aureus* challenge in our study, cause IVD degeneration by inducing regulated cell death and stimulating matrix degradation, chemokine secretion, and changes in IVD cells’ phenotype (75).

The strength of our study lies in the combination of clinical findings and experimental approaches, including the establishment of novel spondylodiscitis-specific *ex vivo* models. We used primary porcine IVD tissue, primary human IVD cells, and neutrophils as well as clinically relevant bacterial isolates for these models, increasing the translational value of our study. However, we also recognize the low patient numbers as the major restricting factor of our study, limiting especially the strength of the histological findings. Additionally, the *ex vivo* models only allow to assess the initial interaction between *S. aureus* and IVD cells but fail to recapitulate a potential detrimental inflammatory role of infiltrating neutrophils into the IVD, which would require a live animal model.

Nevertheless, our findings suggest that *S. aureus* directly interacts with IVD cells and induces a similar inflammatory phenotype in acute spondylodiscitis as found in low-grade chronic IVD degeneration. These findings have important implications and suggest that future therapeutic approaches should explore the potential impact of employing cytokine signaling interference, such as antibody-based treatment, or cell death inhibitors as a complementary therapy to antibiotics in *S. aureus* spondylodiscitis.

## Data availability statement

The raw data supporting the conclusions of this article will be made available by the authors, without undue reservation.

## Ethics statement

The studies involving human participants were reviewed and approved by Cantonal Ethical Research Committee Zurich. The patients/participants provided their written informed consent to participate in this study.

## Author contributions

TAS and AZ conceived the project and were involved in the experimental design. TAS performed most of the experiments and analyzed and compiled the data. FA performed transmission electron microscopy and sample preparation. CA, TCS, NE, and SB collected epidemiological as well as clinical data on spondylodiscitis patients. IH and SD collected human intervertebral discs and provided guidance for cell isolation. EM performed the histology. TAS and FA wrote the first draft of the manuscript. All authors helped in editing the final version of the manuscript and approved it.

## Funding

This work was funded by the SNSF project grant 31003A_176252 (to AZ) as well as the Clinical Research Priority Program of the University of Zurich ‘Precision medicine for Bacterial Infections’ (to AZ and SB).

## Acknowledgments

We would like to thank our patients for participating in clinical and translational research, Ines Kleiber-Schaaf and Andrea Garcete for assistance with histology, Miriam Lipiski for contributing porcine spines, and the Center for Microscopy and Image Analysis, University of Zurich, for support with conventional, confocal laser scanning, and transmission electron microscopy.

## Conflict of interest

The authors declare that the research was conducted in the absence of any commercial or financial relationships that could be construed as a potential conflict of interest.

## Publisher’s note

All claims expressed in this article are solely those of the authors and do not necessarily represent those of their affiliated organizations, or those of the publisher, the editors and the reviewers. Any product that may be evaluated in this article, or claim that may be made by its manufacturer, is not guaranteed or endorsed by the publisher.
