# Supplementary material for: Intervertebral disc cell chondroptosis elicits neutrophil response in Staphylococcus aureus spondylodiscitis
Source: Front Immunol. 2022 Jul 28;13:908211. doi: 10.3389/fimmu.2022.908211 (PMC9366608; doi:10.3389/fimmu.2022.908211)
Supplement: Supplementary file 1 [file DataSheet_1.pdf]

Intervertebral disc cell chondroptosis elicits neutrophil response in *Staphylococcus aureus* spondylodiscitis

Tiziano A. Schweizer[1], Federica Andreoni[1], Claudio Acevedo[1], Thomas C. Scheier[1], Irina Heggli[2,3], Ewerton Marques Maggio[4], Nadia Eberhard[1], Silvio D. Brugger[1], Stefan Dudli[2,5], Annelies S. Zinkernagel[1,5]

[1]Department of Infectious Diseases and Hospital Epidemiology, University Hospital Zurich, University of Zurich, Switzerland
[2]Center of Experimental Rheumatology, University Hospital Zurich and Balgrist University Hospital, University of Zurich, Switzerland
[3]Department of Physical Medicine and Rheumatology, University Hospital Zurich and Balgrist University Hospital, University of Zurich, Switzerland
[4]Department of Pathology and Molecular Pathology, University Hospital Zurich, University of Zurich, Switzerland
[5]Center for applied Biotechnology and Molecular Medicine (CABMM), University of Zurich, Switzerland

Supplemental Material

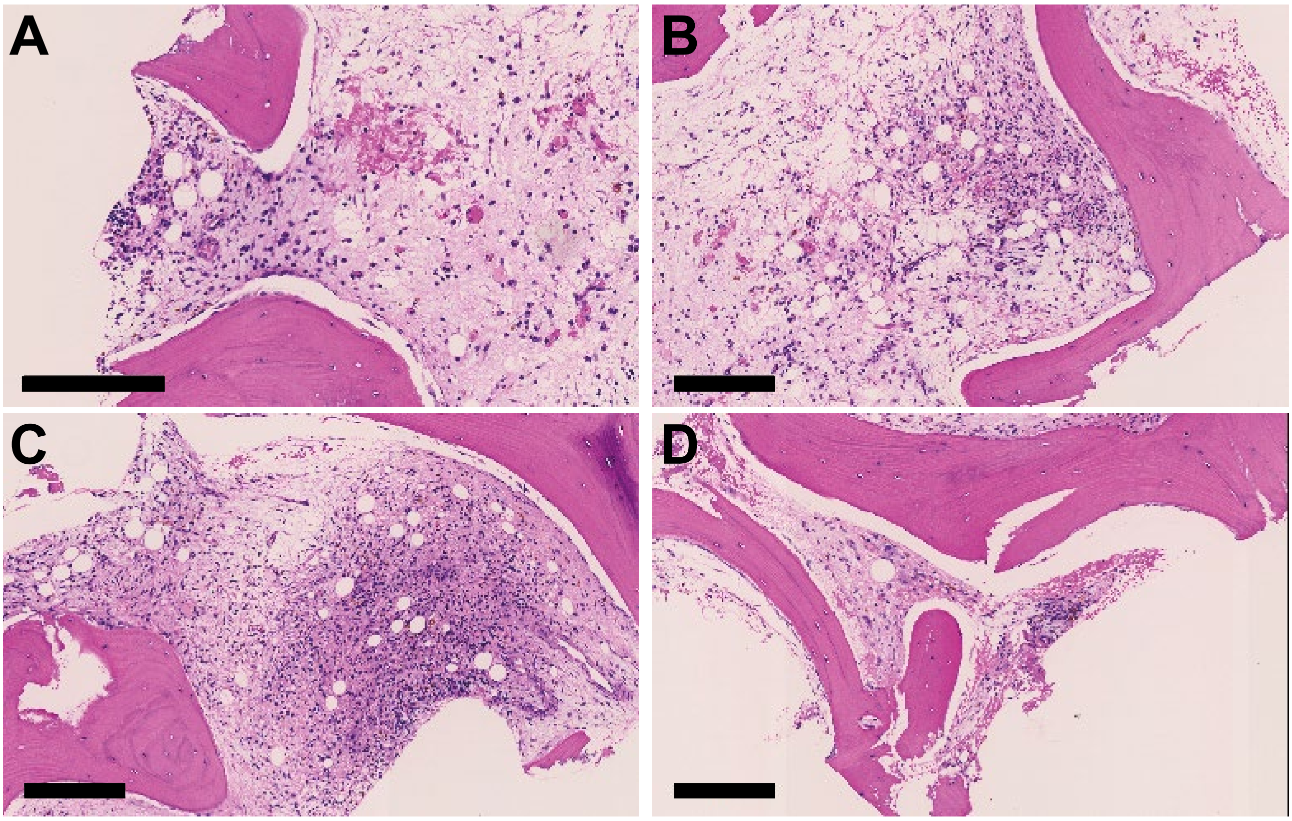

Supplemental Figure 1. (**A-D**) H&E staining of chronic spondylodiscitis case showing presence of lymphocytes and slightly inflamed VB and IVD tissue. Scale bars indicate 200 µm. H&E, Hematoxylin & Eosin; VB, vertebral body; IVD, intervertebral disc.

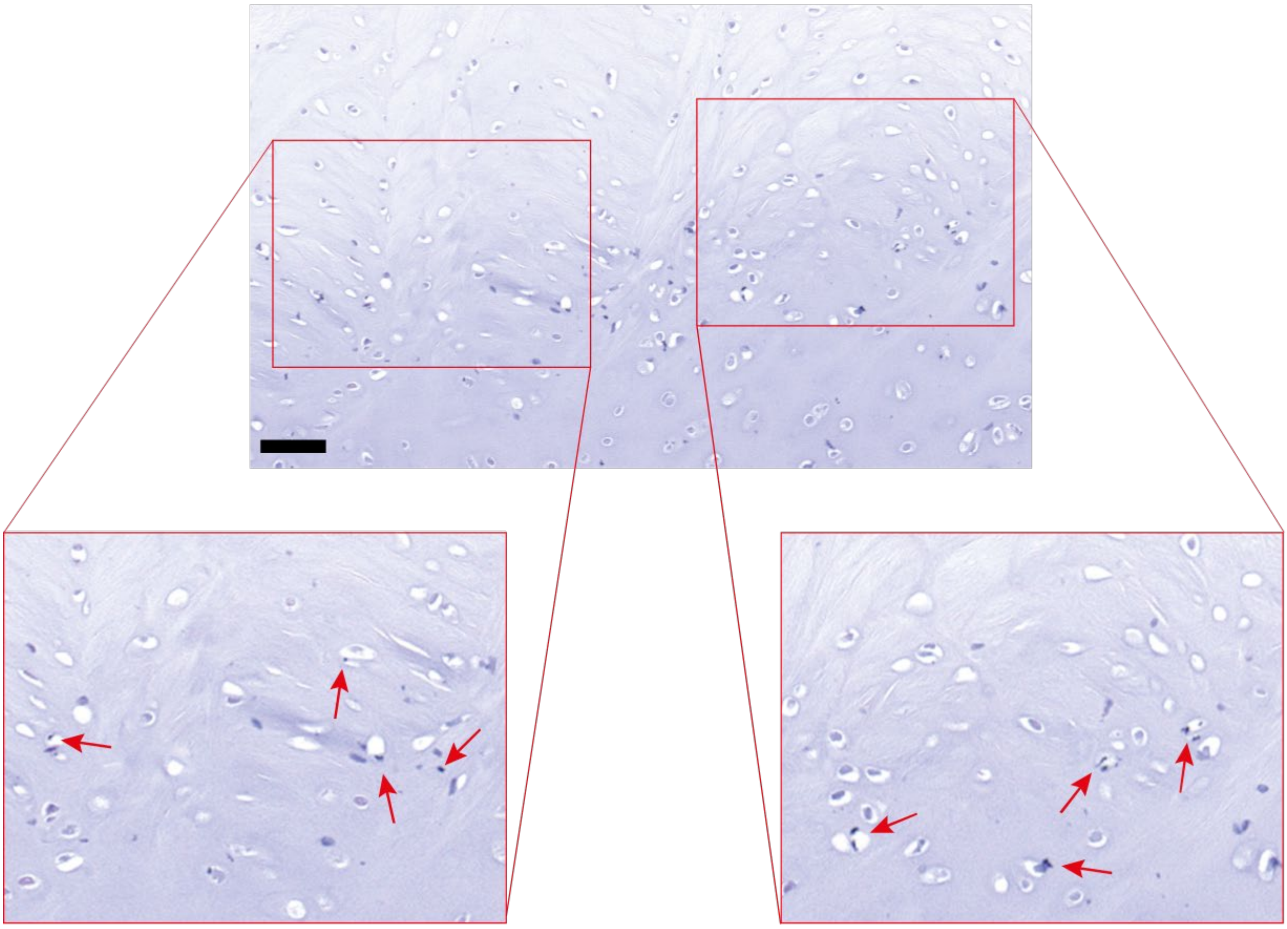

Supplemental Figure 2. Gram staining of *S. aureus* challenged IVD punch, showing the presence of *S. aureus* deep within the IVD punch (red arrows). Scale bar indicates 50 μm. IVD, Intervertebral disc.

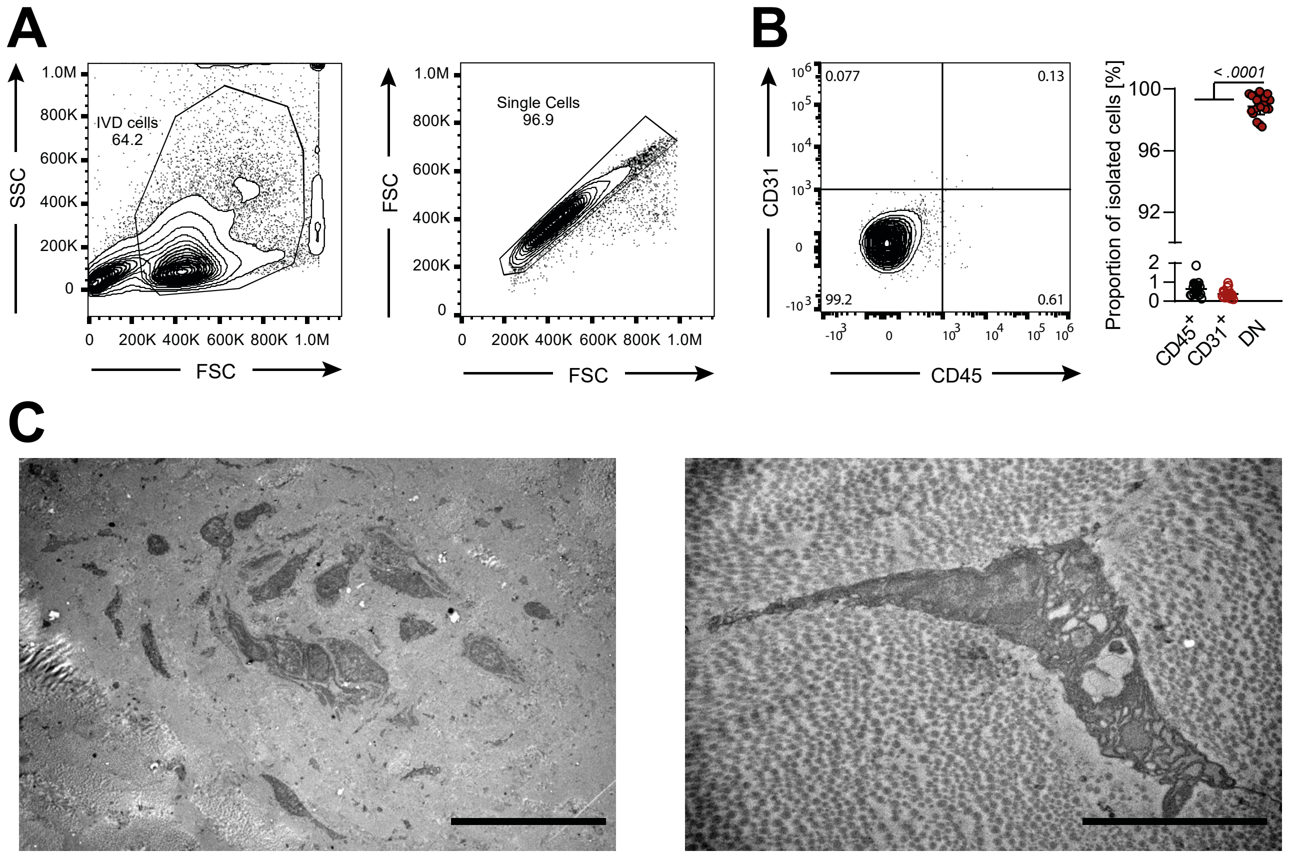

Supplemental Figure 3. (**A**) Gating strategy of IVD cells enzymatically isolated from porcine IVD punches. (**B**) Representative flow cytometry plot and quantification of percentage CD45+, CD31+ or DN cells isolated from porcine IVD punches. (**C**) TEM of IVD cells from unchallenged porcine IVD punches. Scale bars indicate 20 µm (left panel) and 5 µm (right panel). Each dot represents isolated cells from one IVD punch. Statistical analysis was done by One-way ANOVA and Turkey's multiple comparison. IVD, intervertebral disc; DN, double negative; TEM, transmission electron microscopy.

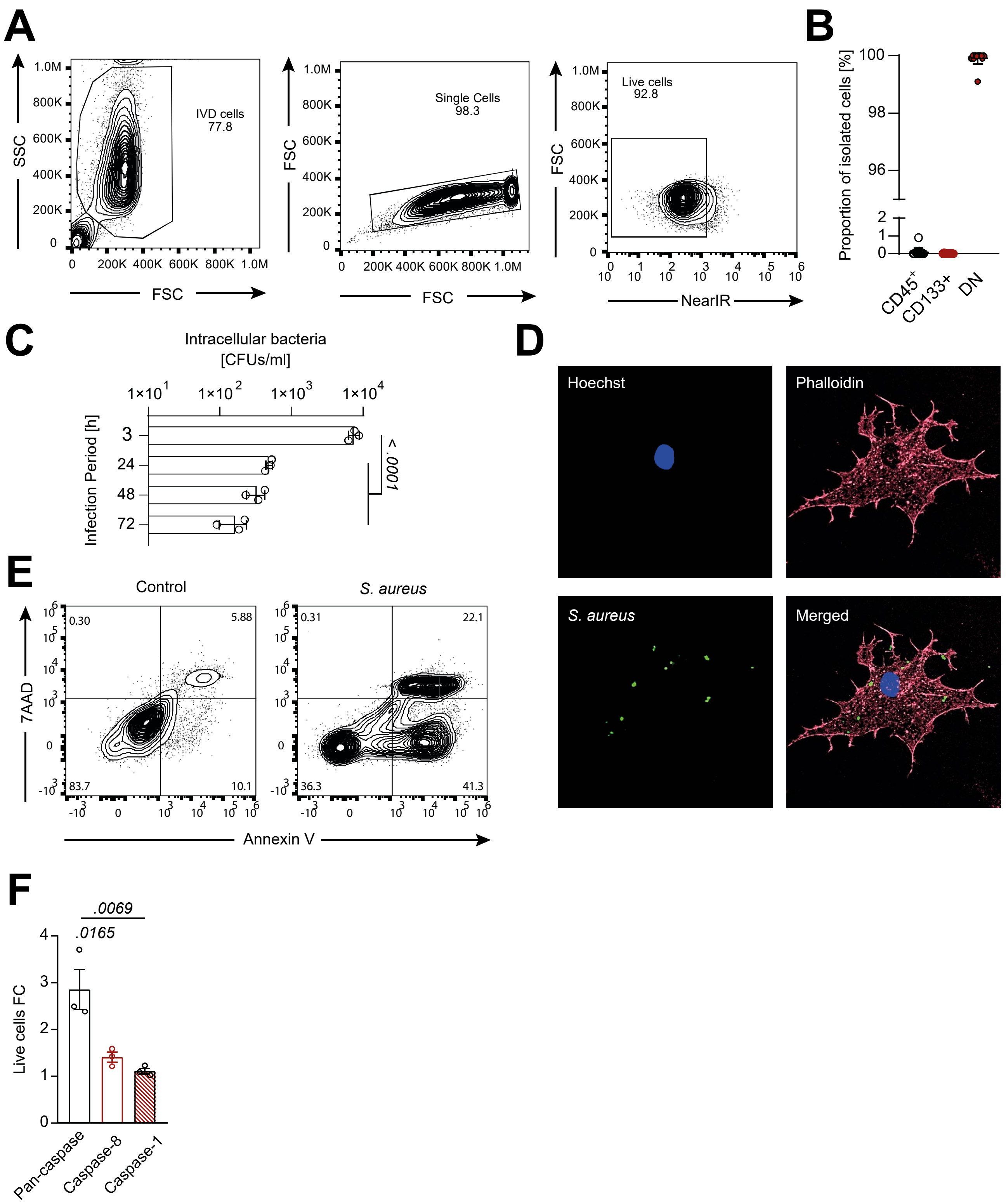

Supplemental Figure 4. (**A and B**) Gating strategy of primary human IVD cells (A) and assessment of purity, by means of absence of CD45+ or CD133+ cells (B). (**C**) Quantification of IVD cells harboring intracellular bacteria over time. (**D**) Representative CLSM micrograph showing IVD cells harboring intracellular *S. aureus*. (**E**) Representative flow cytometry plot of cell death assessment of unchallenged or *S. aureus* challenged IVD cells. (**F**) Assessment of effect on survival of the pan-caspase (Q-VD-OP), caspase-8 (Z-IETD-FMK) and caspase-1 (Z-YVAD-FMK) inhibitors upon *S. aureus* challenge, indicated as FC to untreated IVD cells. Each dot represents one biological

replicate. Statistical analysis was done by one-way ANOVA with Turkey's multiple comparison test. IVD, intervertebral disc; CLSM, confocal laser scanning microscopy; FC, fold change.

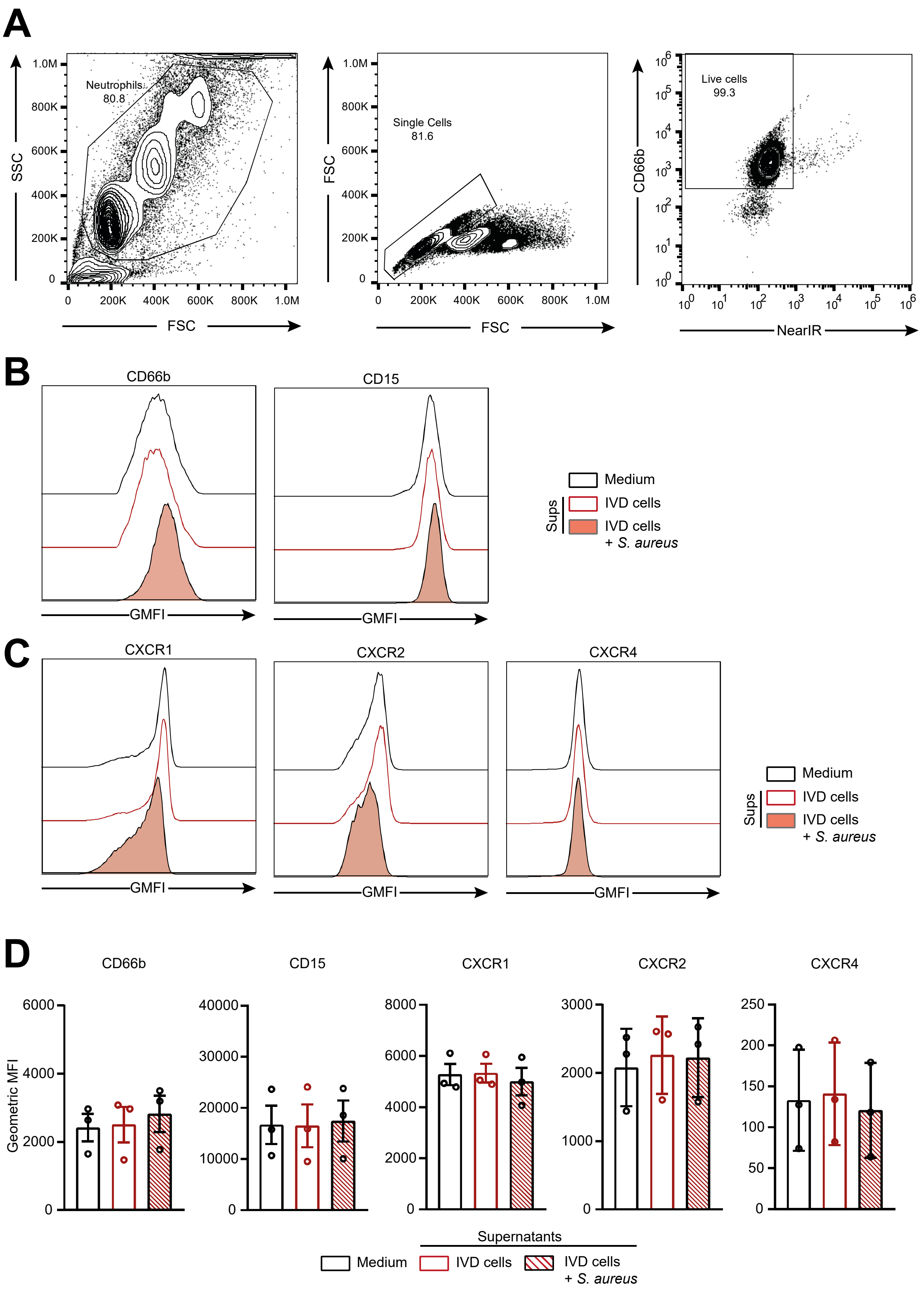

Supplemental Figure 5. (**A**) Gating strategy of primary human neutrophils. (**B and C**) Representative flow cytometry histograms of surface CD66b and CD15 (B) as well as CXCR1,

CXCR2 and CXCR4 expression (C) on neutrophils stimulated with either medium only or supernatant from 24h unchallenged or *S. aureus* challenged primary human IVD cells. (**D**) Quantification of receptor expression on neutrophils stimulated with either medium only or supernatant from 6h unchallenged or *S. aureus* challenged primary human IVD cells. IVD, intervertebral disc.

Supplemental Table 1. ***Staphylococcus aureus*** **strains used in this study.**

Strain	Description	Source/Reference
LS (JE2)	USA300 Laboratory Strain, MRSA	NARSA
#1	Clinical spondylodiscitis Isolate, MSSA (Patient 6)	This study
#2	Clinical spondylodiscitis isolate, MRSA (Patient 7)	This study
#3	Clinical IAI spondylodiscitis isolate, MRSA	This study

LS, laboratory strain; MRSA, Methicillin-resistant *S. aureus*; NARSA, Network on Antimicrobial Resistance in *S. aureus*; MSSA, Methicillin-susceptible *S. aureus*; IAI, Implant-associated infection.
